# Circulating Pentraxin3-Specific B Cells Are Decreased in Lupus Nephritis

**DOI:** 10.3389/fimmu.2019.00029

**Published:** 2019-01-25

**Authors:** Mariele Gatto, Annika Wiedemann, Nadja Nomovi, Karin Reiter, Eva Schrezenmeier, Thomas Rose, Franziska Szelinski, Andreia C. Lino, Sonia Valentino, Anna Ghirardello, Thomas Dörner, Andrea Doria

**Affiliations:** ^1^Unit of Rheumatology, Department of Medicine, University of Padova, Padua, Italy; ^2^Department Medicine, Rheumatology and Clinical Immunology, Charité – Universitätsmedizin Berlin, Berlin, Germany; ^3^Department of Medicine, Nephrology and Medical Intensive Care, Charité – Universitätsmedizin Berlin, Berlin, Germany; ^4^Deutsches Rheuma-Forschungszentrum (DRFZ), Berlin, Germany; ^5^Humanitas Clinical and Research Center, Milan, Italy

**Keywords:** SLE, PTX3^+^ B cells, lupus nephritis, biomarkers, flow-cytometry

## Abstract

**Background:** Pentraxin3 (PTX3) is overexpressed in kidneys of patients developing lupus nephritis (LN). Active LN is associated with reduced anti-PTX3 antibodies. However, abnormalities of B cell differentiation against PTX3 have not been characterized in systemic lupus erythematosus (SLE).

**Objective:** Characterization of PTX3-specific (PTX3^+^) B cells in peripheral blood of SLE patients with or without LN and healthy donors (HD).

**Patients and Methods:** SLE patients without LN, biopsy-proven LN and matched HD were analyzed. Active LN was defined as proteinuria>0.5 g/day or CrCl<60 ml/min/1.73 m^2^ with active urinary sediment. Peripheral B cells were analyzed for direct PTX3 binding by flow cytometry using PTX3 labeled with cyanine 5 (Cy5) and phycoerythrin (PE).

**Results:** Initially, a flow cytometry based assay to identify PTX3^+^ B cells was developed by demonstrating simultaneous binding of PTX3-Cy5 and PTX3-PE. Specificity of B cells was validated by blocking experiments using unlabeled PTX3. We could identify circulating PTX3^+^ B-cells in HD and patients. Notably, LN patients showed a significantly diminished number of PTX3^+^ B cells (SLE vs. LN *p* = 0.033; HD vs. LN *p* = 0.008). This decrease was identified in naïve and memory B cell compartments (naïve: SLE vs. LN *p* = 0.028; HD vs. LN *p* = 0.0001; memory: SLE vs. LN *p* = 0.038, HD vs. LN *p* = 0.011).

**Conclusions:** Decreased PTX3^+^ B cells in LN within the naïve and memory compartment suggest their negative selection at early stages of B cell development potentially related to a decreased regulatory function. PTX3^+^ B cells could candidate for autoantigen-defined regulatory B cells as a striking abnormality of LN patients.

## Introduction

Lupus nephritis (LN) is a severe manifestation of systemic lupus erythematosus (SLE), involving up to two thirds of patients at onset or during disease course (1–3). The inflammatory process in the kidney is driven by both cellular and humoral abnormalities, in particular formation of immune complexes (4–6).

Pentraxin3 (PTX3) belongs to the long pentraxin family, i.e., a superfamily of multimeric evolutionary conserved molecules which are released locally at sites of inflammation (7–12) and are involved in tissue homeostasis, with prominent antimicrobial functions and fine-tuning of inflammation (8–10). It has been proposed as a bridge between innate and adaptive immunity, being endowed with antibody-like properties i.e., the capability to provide opsonization of foreign or apoptotic bodies (9), to modulate antigen presentation and inflammatory responses. Indeed, soluble PTX3 exerts an anti-inflammatory function by sequestering C1q, while when bound to apoptotic debris PTX3 allows C1q fixation and subsequent enhanced activation of the classical complement pathway (8, 10, 13).

Lupus kidney is a source of autoantigens (14, 15), and current evidence supports PTX3 involvement in SLE-driven renal inflammation. Deposits of PTX3 have been characterized in renal samples of several immune-mediated kidney diseases including LN (16–18), where the extent of deposition correlated with proteinuria and renal fibrosis (11, 17, 18).

Moreover, clinical observations showed that SLE patients display high frequencies and titers of anti-PTX3 antibodies (18–20), which are inversely correlated with LN occurrence (18, 19, 21). Furthermore, PTX3 immunization of lupus-prone mice resulted in anti-PTX3 antibody occurrence and was associated with delayed and milder lupus-like nephritis and increased overall and disease-free survival (22), thus providing evidence for an immunomodulatory capacity of anti-PTX3 antibodies.

Despite the interaction of PTX3 with diverse cell types has been described (9, 13, 23), its relationship with B cells remains blurry. Recently, PTX3 released by specialized neutrophils was described to interact with marginal zone (MZ) B cell thereby promoting class-switch from IgM to IgG antibodies (24), however a direct interaction was not described.

In light of the aforementioned observations and open questions, and considering the established role of B cells in LN development (25), it is attractive to speculate that PTX3-specific (PTX3^+^) B cells could bear a regulatory potential in lupus and particularly in LN. Herein, we sought to develop a new method that allows identification and characterization of peripheral PTX3^+^ B cells. Using this methodology, we found PTX3^+^ B cells in SLE patients and healthy donors (HD) which were virtually absent in LN patients. This data suggests that these autoantigen specific B cells may represent a layer of regulation that is lost in LN patients.

## Patients and Methods

Thirty-eight consecutive SLE patients (American College of Rheumatology criteria), including 12 with biopsy-proven LN, and 22 HD were recruited.

Active LN was defined as proteinuria > 0.5 g/day or creatinine clearance < 60 ml/min/1.73 m^2^, evaluated with Cockcroft and Gault formula, with active urinary sediment (3). Accordingly, LN was considered active in 7/12 patients. Demographics of all groups and clinical and therapeutic features of patients are given in Table 1.

**Table 1:** Demographic and therapeutic features of healthy donors and patient groups.

	**Healthy donors**	**Patients**	
		**Non-renal SLE**	**LN**
No.	22	26	12
Mean age ± SD	33.4 ± 8.6	34.6 ± 10.2	41.8 ± 10.37
Female (%)	77.2	84.6	75
Active disease (cSLEDAI≥2) (%)		3/26 (11.5%)	7/12 (58.3%)
cSLEDAI [mean ± SD]		1.14 ± 3.66	4.17 ± 3.86
LN class^*^ **(no.)**			
Proliferative (III or IV)		N/A^**^	7
V or mixed			5
24-h proteinuria (g) [mean ± SD]		N/A	4 ± 1.41
Active urinary sediment^§^ (%)		0	41.7
**CONCOMITANT TREATMENT**			
Oral prednisone (%) [mean daily dosage ± SD]		60.8 [4.04 ± 4.14]	58.3 [3.98 ± 6.05]
HCQ (%)		69.4	58.3
Immunosuppressants (%)		73.9	66.7
MMF		34.7	41.7
MTX		4.3	0
AZA		30.4	25

* International Society of Nephrology/Renal Pathology Society (ISN/RPS) 2003.

** *These patients never underwent kidney biopsy due to lack of renal involvement. § > 5 white blood cells and/or >5 red blood cells/high power field and/or heme-granular/red cell casts. SLE, systemic lupus erythematosus; LN, lupus nephritis; SD, standard deviation; cSLEDAI, clinical SLE disease activity index; HCQ, hydroxychloroquine; MMF, mycophenolate mofetil; MTX, methotrexate; AZA, azathioprine; N/A, not available*.

All subjects gave written informed consent, in accordance with the local ethics committee of the Charité Universitätsmedizin Berlin.

### Whole Blood Lysis

Blood was obtained before any induction therapy for LN in EDTA vacutainer tubes (BD Biosciences, San Jose, CA, USA) and lysed according to the manufacturer's instructions. Briefly, 1 ml of EDTA blood was incubated with 10 ml of lysing solution (Lysing Buffer BD Pharm Lyse™). The obtained cells were washed three times with phosphate-buffered saline/bovine serum albumin (PBS/BSA) (Miltenyi, Germany).

### Staining Procedure and Flow Cytometry

To identify PTX3^+^ B cells, recombinant human purified PTX3 (10) was labeled with either cyanine 5 (Cy5) or phycoerythrin (PE). Tetanus (TT) staining was performed in parallel as control as previously described (24) in order to establish an optical reference range for PTX3^+^ cells. Antigens were labeled at the German Rheumatism Research Centre (DRFZ), Berlin.

For flow cytometric analysis, the following fluorochrome-labeled antibodies were used: PTX3 staining: anti-CD19 Allophycocyanin (APC)-Cy7 (clone SJ25C1, BioLegend), anti-CD20 Brilliant Violet (BV)510 (clone 2H7, BioLegend), anti-CD27 Fluorescein isothiocynate (FITC) (clone M-T271, BD), IgG PECy7 (clone G18-145, BD), IgD Peridinin-Chlorophyll-protein (PerCp) Cy5.5 (clone IA6-2, BD), anti-CD3/anti-CD14 Pacific Blue (PacB) (clone UCHT1/M5E2, BD). TT staining: anti-CD19 PECy7 (clone SJ25C1, BD), anti-CD20 BV510 (clone 2H7, BioLegend), anti-CD27-FITC, IgD PerCp Cy5.5 (clone IA6-2, BD), anti-CD3/anti-CD14 PacB (clone UCHT1/M5E2, BD), anti-CD38 APC-Cy7 (clone HIT2, BioLegend).

**Figure 1:**
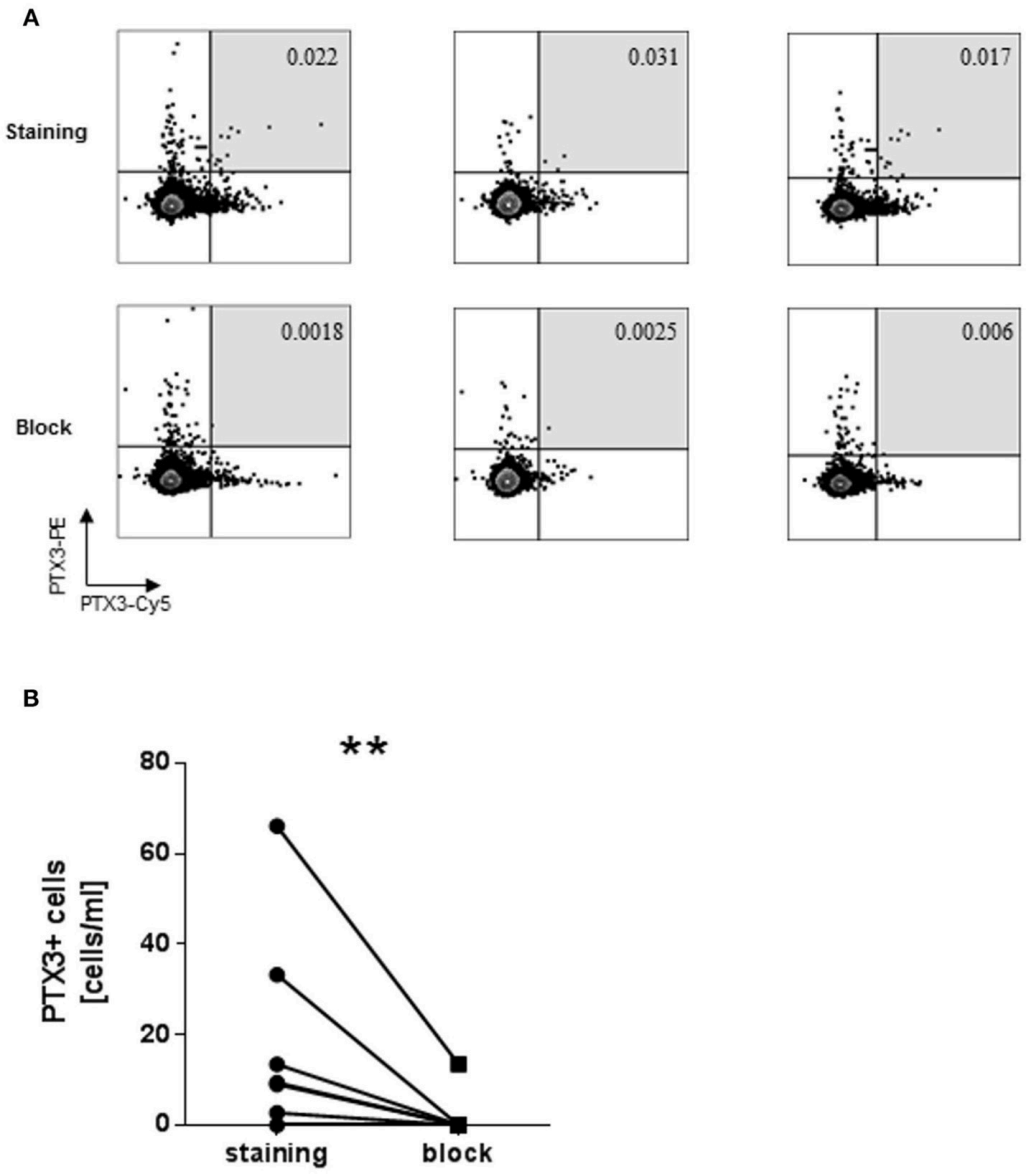
Identification of PTX3+ B cells among CD19+CD20+ B cells. **(A)** Three representative dot plots of the PTX3-specific B cells before and after blocking with unlabeled PTX3. Only B cells staining positive for both PTX3-Cy5 and PTX3-PE were considered (light gray square). **(B)** Quantification of PTX3 binding among B cells before and after blocking of PTX3. Cy5, cyanin 5; PE, phycoerythrin. ^**^ < 0.01.

PTX3^+^B cells were identified as B cells binding both PTX3-PE and PTX3-Cy5 (Figure 1).

TT-specific B cells were identified by the binding of TT-Cy5. Specificity of this binding was checked in parallel stainings performed in our laboratory.

After Fc receptor blocking (Miltenyi Biotec, Germany), stainings were performed in the dark at 4°C for 15 min, followed by two washing steps with PBS/BSA and centrifugation for 5 min at 4°C and 330 × g. Stained cells were analyzed by flow cytometry using a FACS Canto II flow cytometer (BD, USA).

B cell subsets were defined as naïve (single CD3^−^CD14^−^Dapi^−^CD19^+^CD20^+^CD27^−^), memory (single CD3^−^CD14^−^Dapi^−^CD19^+^CD20^+^CD27^+^) and plasmablasts (single CD3^−^CD14^−^Dapi^−^CD19^+^CD27^hi^CD20^−^).

The gating strategy is exemplarily shown in Supplementary Figure 1. Absolute numbers of B subpopulations were calculated by using the absolute number of B cells/μl retrieved with Multitest 6-color TBNK analysis (BD, USA) according to the manufacturer's protocol.

### Data Analysis and Statistics

Samples included in analyses had at least 1 × 10^6^ events with a minimum threshold for CD19^+^ cells of 50,000 events.

Flow cytometric data was analyzed by FlowJo software 7.6.5 (TreeStar, Ashland, OR, USA). GraphPad Prism Version 5 (GraphPad software, San Diego, CA, USA) was used for statistical analysis. To test for significance, non-parametric tests were used.

## Results

### Identification of PTX3^+^ B Cells in Peripheral Blood of Patients and Controls

Initially, we developed a flow cytometric method to identify and quantify PTX3^+^ B cells in human peripheral blood. B cells (CD3^−^CD14^−^CD19^+^CD20^+^) able to bind simultaneously PTX3-Cy5 and PTX3-PE were considered specific (Figure 1A) (gated as shown in Supplementary Figure 1). The specificity of PTX3 binding was further confirmed by blocking with unlabeled PTX3 prior to staining (Figures 1A,B). Blocking of single positive PE or Cy5-PTX3 B cells could not be efficiently performed (Supplementary Figure 2), thus these cells were not included in the present analysis.

### PTX3^+^B Cells Are Decreased in LN Patients vs. SLE and HD

**Figure 2:**
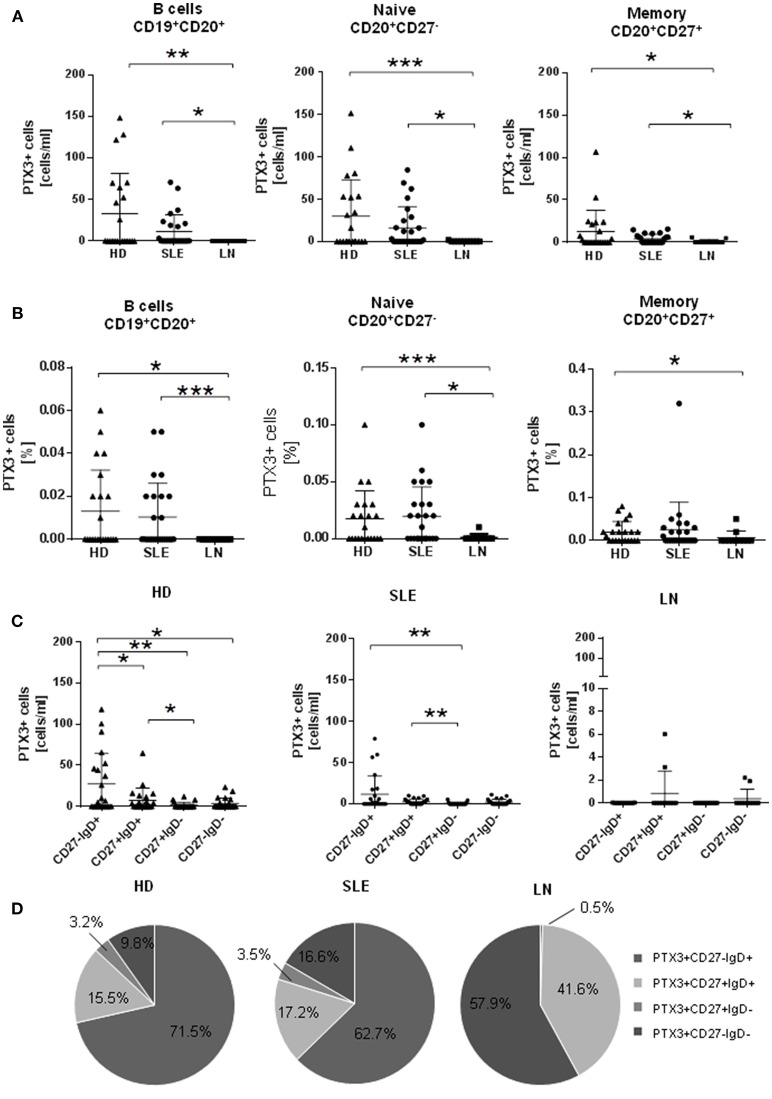
PTX3^+^ B cells are decreased in patients with lupus nephritis and are mainly confined to CD27^−^IgD^+^ B cells. **(A)** Absolute numbers of PTX3^+^ B cells (cell/mL) within (left) total; (middle) naïve or (right) memory B cells in HD (*n* = 22) and SLE (*n* = 26) and LN (*n* = 12) patients. **(B)** Frequencies of PTX3^+^ B cells (left), naïve (middle), and memory (right) are decreased in LN (*n* = 12) in comparison with HD (*n* = 22) and SLE (*n* = 26). **(C)** Distribution of CD27 and IgD expression by PTX3^+^ B cell subsets are shown. Enrichment in the naïve pool with decreases in the other subsets was found in HD (*n* = 22) and SLE (*n* = 26), but not in LN (*n* = 12). **(D)** Pie charts of percentages of PTX3^+^ CD27IgD subsets within the PTX3^+^ B cell pool are consistent with distribution of absolute numbers. Mann-Whitney *U*-test (^*^ < 0.05, ^**^ < 0.01, ^***^ < 0.001). SLE, systemic lupus erythematosus; HD, healthy donors; LN, lupus nephritis; PTX3 pentraxin3.

LN patients showed the lowest absolute numbers of PTX3^+^ B cells among total peripheral B cells, which were also significantly decreased in comparison with HD and non-renal SLE [mean ± standard deviation (SD) cells/ml: LN 0.023 ± 0.027 vs. HD 33.09 ± 48.15, *p* = 0.008; LN 0.023 ± 0.027 vs. non-renal SLE 12.53 ± 20.24, *p* = 0.033] (Figure 2A, left).

Analyses of B cell subsets confirmed a substantial decrease in absolute numbers of both naïve (CD20^+^CD27^−^) and memory (CD20^+^CD27^+^) PTX3^+^ B cells in LN compared to HD and non-renal SLE patients (mean ± SD naïve/ml: LN 0.18 ± 0.58 vs. HD 30.12 ± 42.96, *p* = 0.0001; LN SLE 0.18 ± 0.58 vs. non-renal 16.22 ± 24.88, *p* = 0.028; mean ± SD memory/ml: LN 0.97 ± 2.18 vs. HD 12.75 ± 24.88, *p* = 0.011; LN 0.97 ± 2.18 vs. non-renal SLE 4.07 ± 5.21, *p* = 0.038) (Figure 2A, middle and right).

Moreover, the frequencies of PTX3^+^ B cells and B cell subsets were decreased in LN (Figure 2B), while there was no significant difference between HD and non-renal SLE.

Of note, no difference in PTX3^+^ naïve and memory compartment was identified between active and inactive LN (data not shown), suggesting that the actual decrease in LN is not related to disease activity, rather mirroring a characteristic of LN.

We detected nearly no circulating PTX3^+^ plasmablasts (CD27^hi^CD20^−/low^) in whole blood sampled for the majority of donors, where a minimum of 1 × 10^6^ events was retrieved from each sample. These cells were absent even when a larger amount of cellular events (27 × 10^6^) from an SLE patient among a total of 7,648 plasmablasts was analyzed.

### Circulating PTX3^+^ B Cells Reside Mainly Within Naïve (CD20^+^CD27^−^IgD^+^) B Cells With a Similar Distribution Among HD and Non-renal SLE Patients

Using CD27 and IgD as surface markers, we further subdivided B cell subpopulations. We found that the majority of circulating PTX3^+^B cells resided among the CD20^+^CD27^−^IgD^+^ mature pre-switch naïve subset, followed by a lower number of CD20^+^CD27^+^IgD^+^ B cells (Figures 2C,D), likely representing pre-switched memory B cells whose origin is still debated (26). This distribution remained consistent among HD and non-renal SLE patients (Figure 2C, left and middle), while LN patients did not show any difference among B cell subsets (Figure 2C, right). Proportions of PTX3^+^ CD27IgD B cell subsets in relationship to the whole PTX3^+^ B cell pool are shown in Figure 2D.

## Discussion

In this study, we aimed at characterizing the distribution of an PTX3^+^ B cells in peripheral blood of SLE patients, focusing on potential differences between LN and non-renal SLE. Most interestingly, our results show that LN patients bear strikingly reduced amounts of circulating PTX3^+^ B cells both in the naïve and memory compartment vs. HD and non-renal SLE. This finding was observed regardless of LN activity, suggesting a characteristic divergence between patient subsets.

Moreover, the current results indicate that selection of PTX3 binding B cells appears to be defective in LN based on the analysis of sole PTX3^+^ B cells and B cell subsets which were able to bind specifically PTX3 molecules using two different dyes blockable by unlabeled antigen. The observation that most circulating PTX3^+^B cells belong to CD27^−^IgD^+^ and to CD27^+^IgD^+^ subsets is not surprising, since PTX3 is a self-antigen without enhanced antigenicity. In this context, lack of detectable PTX3^+^ plasmablasts is consistent with the notion that the B and plasma cell compartments underlie independent regulations (27–30), thus at this point it is not possible to establish a direct link between these specific PTX3-B cell subsets and the production of anti-PTX3 antibodies.

Notably, by virtue of its octameric structure, PTX3 is able to bind diverse molecules (12, 31, 32) and either living or apoptotic cells (9, 13, 23). More recently, the binding of PTX3 to marginal zone (MZ) B cells was described which promoted production of IgM and IgG antibodies, thus suggesting that PTX3 is involved in the regulation of B cell differentiation or potentially in B helper function (24). So far, however, the binding site of PTX3 other than Fcɤ receptors or Toll-like receptors (23, 24) has not been fully delineated.

It is well-known that regulatory B cell function (both in numbers and functionality) is impaired in SLE (33, 34) and a stable decrease of naïve and memory PTX3^+^B cells in LN patients may hint to an early negative selection or depletion of this specificity in LN. While previous pre-clinical and clinical data showed that anti-PTX3 autoantibodies carry a LN protective potential, it remains to be shown whether this also applies to a potential immunomodulatory function of PTX3^+^ B cells. Alternatively, and not mutually exclusively, the abundance of naïve PTX3^+^ B cells in non-renal SLE may represent polyreactive B cells, which is a common feature of SLE (35, 36), and may give rise to antibody secreting cells *via* an extrafollicular pathway (37). This would be consistent with the progressive decrease in PTX3^+^B cell subsets from naïve, pre-switch to post-switch memory cells. The current finding of a similar level and distribution of PTX3^+^ B cells in non-renal SLE and HD has some implications. First, their absence in LN suggests that their presence has relevance to protect the kidney. Second, selection processes leading to PTX3^+^ B cell subsets might be intact in SLE as observed for HD. Alternatively and related to the proposed protective function, non-renal SLE patients are able to generate a sufficient amount of PTX3^+^ B cells which could be involved in immune protection being more compromised in LN. As such, the absence of circulating PTX3^+^ B cells in LN may hamper the kidney-protective effect provided by PTX3 specific autoantibodies.

Our study is the first addressing the distribution of autoantigen-specific B cells in lupus, focusing on a molecule of emerging importance, through an original method of selection and verification of autoantigen-specific B cells, which can be thereby identified from peripheral blood of patients or controls with a high level of certainty and in the absence of stimulation.

In summary, this study developed a method to reliably identify PTX3-specific B cells and their subsets. Application of this technology allowed the identification of an abnormal distribution of PTX3^+^ B cells with their absence in LN and a very similar profile among controls and non-renal SLE. To which extent these findings relate to a potential immune regulatory role or protective function of this specificity as well as potential clinical applications in diagnostics or therapeutics remains to be delineated.

## Author Contributions

TD, AD, and MG conceived the core idea of the study. KR, AW, AL, FS, and MG established the technology and experimental conditions. NN, AG, and SV provided antigen supply. ES, TR, KR, NN, and MG all contributed to patient recruitment. MG and KR performed laboratory and statistical analysis. MG wrote the manuscript. TD, AD, AL, AW, and AG provided revisions and all authors approved the final version.

### Conflict of Interest Statement

The authors declare that the research was conducted in the absence of any commercial or financial relationships that could be construed as a potential conflict of interest.

## Supplementary Material

Supplementary Figure 1 Representative gating strategy of peripheral B cells according to FSC and SSC characteristics and further identification on their surface expression of CD19, CD20, and subsets according to CD27 surface expression. FSC, forward scatter; SSC, sideward scatter.

Supplementary Figure 2 Quantification of PTX3 binding among B cells that only bind either PTX3-PE **(A)** or PTX3-Cy5 **(B)** before and after blocking of PTX3 is not significant (Wilcoxon rank test). PTX3, pentraxin 3; Cy5, cyanin 5; PE, phycoerythrin.
