# Supplementary figures and images for: Circulating Pentraxin3-Specific B Cells Are Decreased in Lupus Nephritis
Source: Front Immunol. 2019 Jan 25;10:29. doi: 10.3389/fimmu.2019.00029 (PMC6355680; doi:10.3389/fimmu.2019.00029)

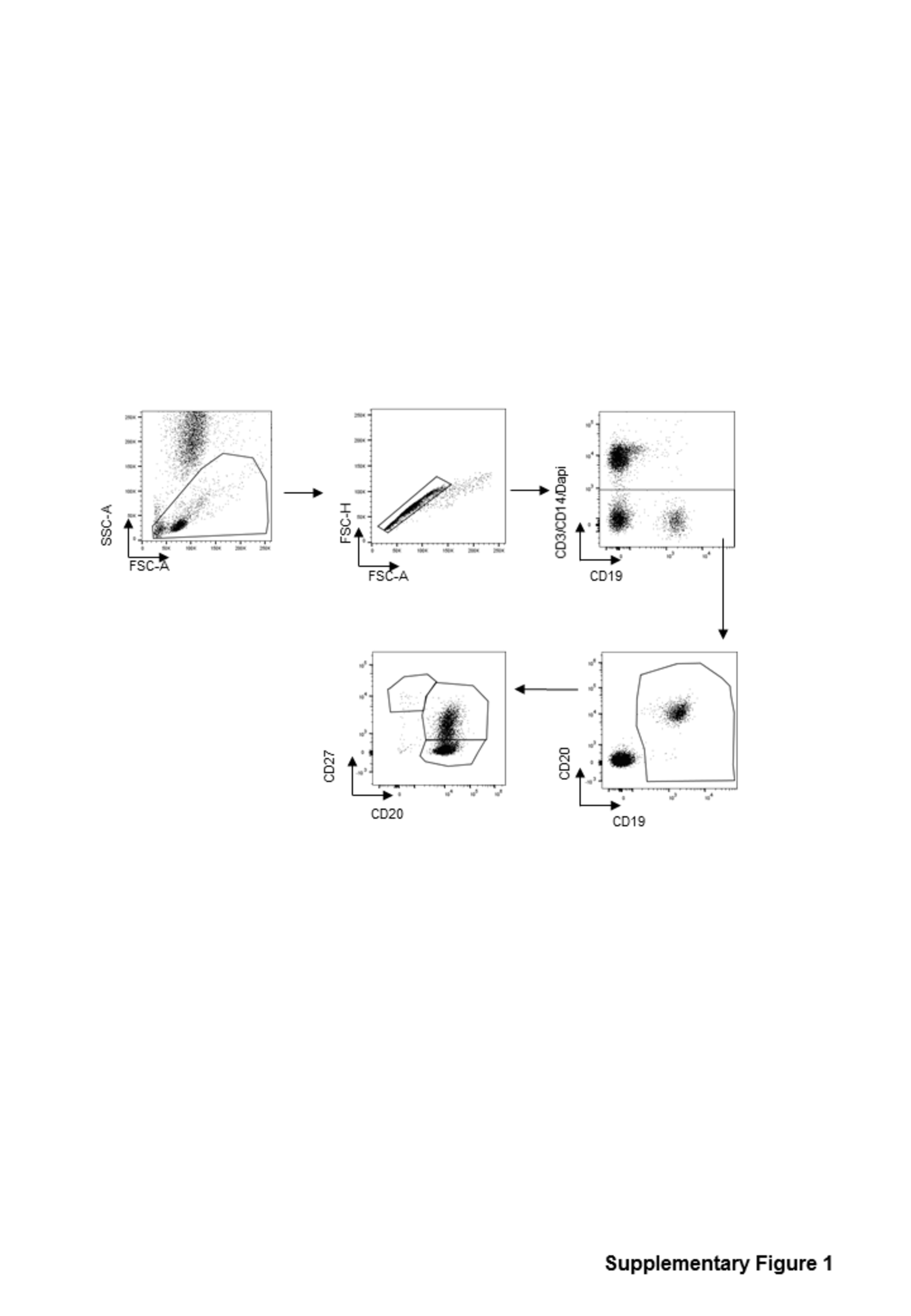

Supplement: Supplementary Figure 1 — Representative gating strategy of peripheral B cells according to FSC and SSC characteristics and further identification on their surface expression of CD19, CD20, and subsets according to CD27 surface expression. FSC, forward scatter; SSC, sideward scatter. [file Image_1.TIF]

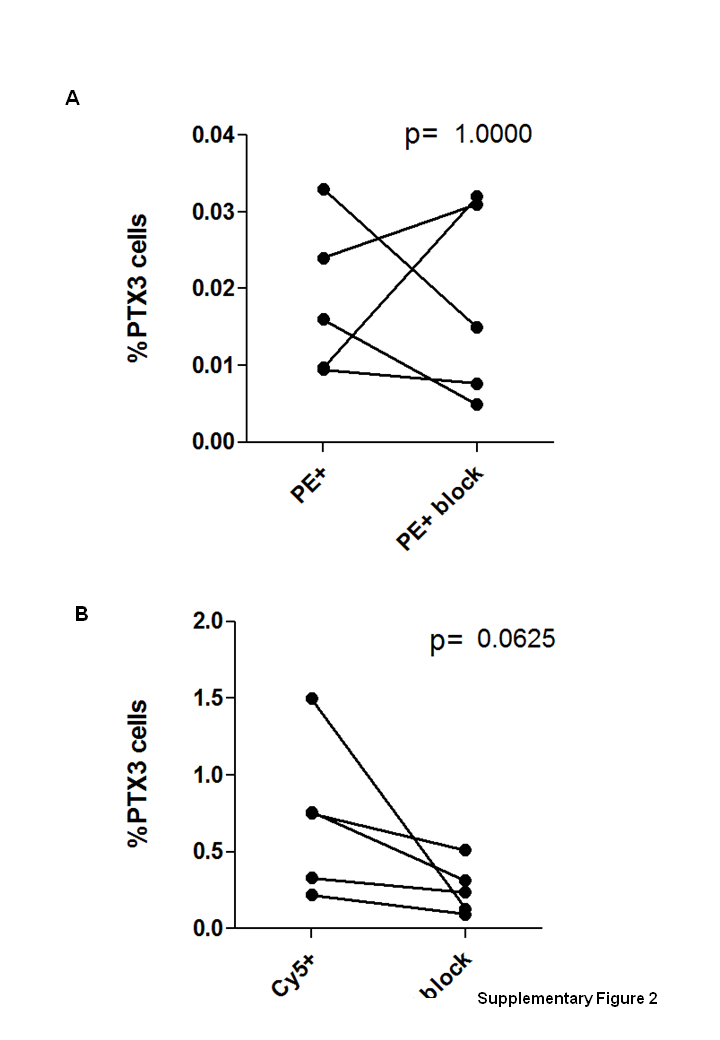

Supplement: Supplementary Figure 2 — Quantification of PTX3 binding among B cells that only bind either PTX3-PE (A) or PTX3-Cy5 (B) before and after blocking of PTX3 is not significant (Wilcoxon rank test). PTX3, pentraxin 3; Cy5, cyanin 5; PE, phycoerythrin. [file Image_2.TIF]
